# Resequencing and comparison of whole mitochondrial genome to gain insight into the evolutionary status of the Shennongjia golden snub‐nosed monkey (SNJ *R. roxellana*)

**DOI:** 10.1002/ece3.3011

**Published:** 2017-05-15

**Authors:** Yanyun Hong, Hairui Duo, Juyun Hong, Jinyuan Yang, Shiming Liu, Lianghui Yu, Tuyong Yi

**Affiliations:** ^1^ College of Plant Protection of Hunan Agricultural UniversityChangshaChina; ^2^ Hunan Provincial Key Laboratory for Biology and Control of Plant Diseases and Plant PestsChangshaChina; ^3^ Nature Reserve College of Beijing Forestry University, BeijingBeijingChina; ^4^ Orient Science & Technology College of Hunan Agricultural UniversityChangshaChina; ^5^ Hubei Province Key Laboratory of Conservation Biology of Shennongjia Golden MonkeyMuyuChina

**Keywords:** divergence ages, mitochondrial genome (mtDNA), next‐generation sequencing, phylogenetic analyses, *Rhinopithecus roxellana*, Shennongjia *Rhinopithecus roxellana*

## Abstract

Shennongjia *Rhinopithecus roxellana* (SNJ *R. roxellana*) is the smallest geographical population of *R*. *roxellana*. The phylogenetic relationships among its genera and species and the biogeographic processes leading to their current distribution are largely unclear. To address these issues, we resequenced and obtained a new, complete mitochondrial genome of SNJ *R. roxellana* by next‐generation sequencing and standard Sanger sequencing. We analyzed the gene composition, constructed a phylogenetic tree, inferred the divergence ages based on complete mitochondrial genome sequences, and analyzed the genetic divergence of 13 functional mtDNA genes. The phylogenetic tree and divergence ages showed that *R. avunculus* (the Tonkin snub‐nosed monkey) was the first to diverge from the *Rhinopithecus* genus ca. 2.47 million years ago (Ma)*. Rhinopithecus bieti* and *Rhinopithecus strykeri* formed sister groups, and the second divergence from the *Rhinopithecus* genus occurred ca. 1.90 Ma. *R. roxellana* and *R. brelichi* diverged from the *Rhinopithecus* genus third, ca. 1.57 Ma. SNJ *R. roxellana* was the last to diverge within *R. roxellana* species in 0.08 Ma, and the most recent common ancestor of *R. roxellana* is 0.10 Ma. The analyses on gene composition showed SNJ *R. roxellana* was the newest geographic population of *R. roxellana*. The work will help to develop a more accurate protection policy for SNJ *R. roxellana* and facilitate further research on selection and adaptation of *R. roxellana*.

## 1 INTRODUCTION

**Figure 1:**
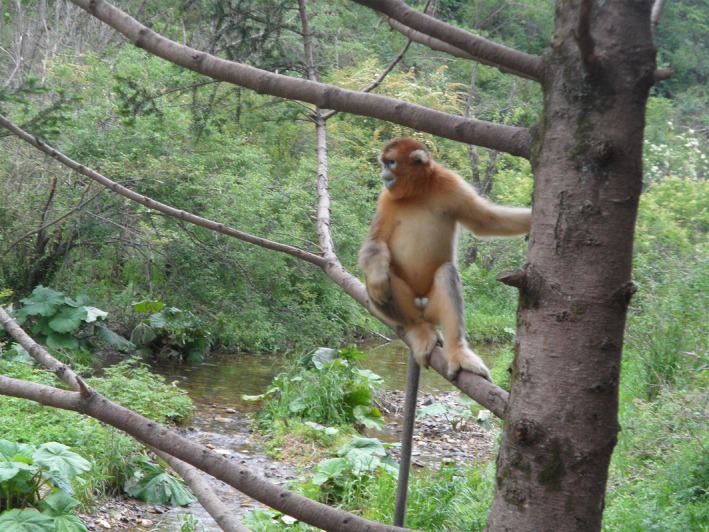
The photograph of *Rhinopithecu roxellana* in Shennongjia National Nature Reserve. It would be used as the graphical table of contents

The *Rhinopithecus* genus (snub‐nosed monkeys) has five species, including three species that only live in China, the Sichuan golden monkey (*Rhinopithecus roxellana*), the Guizhou golden monkey (*Rhinopithecus brelichi*), and the Yunnan golden monkey (*Rhinopithecus bieti*). One species lives in North Vietnam, the Tonkin snub‐nosed monkey (*Rhinopithecus avunculus*; Le & Covert, 2010; Yu, Wang, Ting, & Zhang, 2011). The Myanmar golden monkey (*Rhinopithecus strykeri*) was reported in Myanmar (Burma) but also exists in the contiguous forests in Nujiang Prefecture, China (Geissmann et al., 2011; Liedigk et al., 2012). *Rhinopithecus roxellana* was distributed widely in China during the Pleistocene; however, today, wild *R. roxellana* populations only exist in three isolated mountainous regions, including the Minshan and Qionglai mountains in Sichuan and Gansu provinces (SG), the Qinling mountains in Shaanxi province (QL), and the Shennongjia National Nature Reserve (SNJ) in Hubei province. *Rhinopithecus roxellana* is classified as an endangered species by the World Conservation Union and is protected at Level I by law of the People's Republic of China on the protection of wildlife (Le, 2015; Pan et al., 2009; Zhou et al., 2014). Due to the distinct appearance of *R. roxellana*, a beautiful golden coat and snub nose, and its rarity, *R. roxellana* is an endangered arboreal primate icon in China (Figure 1). *R. roxellana* has a population of approximately 22,000, including approximately 16,000 individuals from the SG population, approximately 5,500 individuals from the QL population, and approximately 1,000 individuals from the SNJ population (Chang, Liu, Yang, Li, & Vigilant, 2012; Yang et al., 2012).

The phylogenetic relationships of the *Rhinopithecus* genus have been studied (Karanth, Singh, Collura, & Stewart, 2008; Li et al., 2010; Osterholz, Walter, & Roos, 2008; Perelman et al., 2011; Sterner, Raaum, Zhang, Stewart, & Disotell, 2006); however, debates on the origin of *R. roxellana* are still ongoing. Wang, Jiang, and Li (1998) found some morphological differences among the *R. roxellana* specimens from Sichuan‐Gansu (SG), Shanxi Qinling (QL), and Hubei Shennongjia (SNJ) and classified the SG, SNJ, and QL populations as *R. roxellana* ssp. *roxellana*,* R. roxellana* ssp. *hubeiensis*, and *R. roxellana* Qinlingensis, respectively (Li et al., 2007). Pan et al. (2009) proposed two hypotheses for the origin of *R. roxellana*. The mono‐origin hypothesis suggested that the QL and SNJ populations originated from the SG population, while the multiorigin hypothesis insisted that the SG population evolved from a fusion of the QL and SNJ populations. Nevertheless, neither of these two hypotheses can perfectly explain the evolutionary relationships among these three populations. The Shennongjia National Nature Reserve is the most eastern habitat of *R. roxellana* in China, and SNJ *R. roxellana* has the smallest *R. roxellana* population. To protect the SNJ *R. roxellana* population, it is particularly important to study the genetic evolution and the genetic diversity of SNJ *R. roxellana*. Analyses of the complete mitochondrial DNA control region suggested that the QL and SNJ populations originated from the SG population, but there was no gene flow between the SNJ and SG populations (Luo, Liu, Pan, Zhao, & Li, 2012). In contrast, the structure analyses of 16 microsatellite loci showed that the SNJ population consisted of two groups and that the SNJ population originated from the SG and QL populations (Chang, Liu, Yang, et al., 2012). Moreover, some studies showed SNJ population had the lowest diversity (Chang, Luo, Liu, et al., 2012; Chang, Liu, Yang, et al., 2012; Luo et al., 2012), other studied showed SNJ population had rich genetic diversity (He, Zhang, Peng, Li, & Li, 2010). However, until now the phylogenetic relationship and genetic diversity of SNJ *R. roxellana* is still unclear.

Mitochondrial DNA (mtDNA) has been widely used as a molecular marker in phylogenetic and phylogeographic studies of the *Rhinopithecus* genus and other primates because it is high mutation and substitution rates, low effective population size, high copy number, rare gene recombination, maternal transmission, and easy accessibility (Finstermeier et al., 2013; Kolleck et al., 2013; Liedigk et al., 2012; Yang et al., 2012; Yu et al., 2011; Zhang, 1998). Mitochondrial genes/fragments such as the D‐loop, Cytochrome b (Cytb), tRNA, and NADH gene have been used for phylogenetic studies on genetic divergence and phylogenetics of the *R. roxellana* species (Kolleck et al., 2013; Le, 2015; Luo et al., 2012; Sterner et al., 2006; Zhang, 1998). The complete mitochondrial genomes were used to analyze primate phylogenetic relationships and divergence date (Pozzi et al., 2014). Moreover, the complete mitochondrial genomes are effectively used as the molecular markers in molecular ecology, population genetics, conservation genetics, and evolutionary biology (Ekblom & Galindo, 2011; Morin et al., 2010; Yann & Juan, 2010). For example, complete mitochondrial genomes have been used to solve many evolutionary questions in humans (Green, Malaspinas, & Krause, 2008; Krause et al., 2010) and to investigate the evolutionary histories of endangered and enigmatic species, such as mammoths, brown bears, aurochs, Tasmanian tigers, and polar bears (Driscoll, Yamaguchi, & Bar, 2009; Edwards et al., 2010; Lindqvist et al., 2010; Miller et al., 2009, 2012; Morin et al., 2010).

In this study, we used the Illumina Hi‐seq2000 platform to resequence the complete mitochondrial genome of SNJ *R. roxellana*. Furthermore, we analyzed the mitochondrial genomic divergence and phylogenetic relationships within the *R. roxellana* species and the *Rhinopithecus* genus to determine whether *R. roxellana* is a monotypic or polytypic species and assess the divergence ages and evolutionary status of the SNJ population within *R. roxellana*.

## 2 MATERIALS AND METHODS

### 2.1 Ethics statement

The research complied with protocols approved by the Forestry Ministry of Hubei Province, China, and abided by the legal requirements of China. Permits to collect samples were provided by the Shennongjia National Nature Reserve, and the staff of the Shennongjia National Nature Reserve helped to collect samples and supported the study. The research was conducted according to animal care regulations and the principles of the American Society of Primatologists.

### 2.2 Sample information

For sequencing, we collected a frozen liver sample of a three‐month‐old male *R. roxellana* who was from Shennongjia National Nature Reserve in China and killed during a male takeover in August 2012. His corpse was immediately stored at −20°C after death.

### 2.3 DNA extraction, short‐read sequencing, de novo assembly and annotation

In the study, we resequenced the mitochondrial genome of SNJ *R. roxellana*. Firstly, we extracted pure liver mitochondria using a tissue mitochondria isolation kit (Beyotime Biotech Inst., Beijing, China), and then, mtDNA was extracted using the blood DNA kit (E.Z.N.A.; Omega, USA). The extract was prepared for both Sanger sequencing and next‐generation sequencing (NGS). One part of the mtDNA was used to construct a paired‐end sequencing library with an insertion size of approximately 500 base pairs (bp) and was sequenced by NGS with Illumina Hi‐seq2000. We prepared the libraries as follows: 200–500 ng DNA is fragmented by Covaris. The fragmented DNA is combined with End Repair Mix, incubate at 20°C for 30 min. Purify the end‐repaired DNA with QIAquick PCR Purification Kit (Qiagen) and then add A‐Tailing Mix, incubate at 37°C for 30 min. Combine the purified Adenylate 3′ Ends DNA, Adapter and Ligation Mix, incubate the ligation reaction at 20°C for 15 min. Adapter‐ligated DNA is selected by running a 2% agarose gel to recover the target fragments. Purify the gel with QIAquick Gel Extraction kit (Qiagen). Several rounds of PCR amplification with PCR Primer Cocktail and PCR Master Mix are performed to enrich the Adapter‐ligated DNA fragments. Then, the PCR products are selected by running another 2% agarose gel to recover the target fragments. Purify the gel with QIAquick Gel Extraction kit (Qiagen). The remainder mtDNA was used to perform a PCR amplification by the primers MT_(15,928–16,543)_ and Mt_(7–535)_ (Table 1) and general sequencing to link the gaps between scaffolds. The PCR products were preliminarily confirmed on a 1.0% agarose gel, sequenced directly in two reactions with forward and reverse primers by Sanger sequencing and then spliced with contigs or scaffolds using the ContigExpress software package.

**Table 1:** The information on the primers

	Sequence (5′–>3′)	Tm	Product length
MT_(15,928–16,543)_ forward primer	ATTTAGTCTGGCTTTTGAAG	60.46	615 bp
Reverse primer	GATAACAGCGCAATCCTATTC	59.48	
Mt_(7–535)_ forward primer	ATCGACATAGGGTTTACGA	59.01	528 bp
Reverse primer	CTTAAAACCTTCAACCTCC	60.14	

We used *SOAP* de novo to assemble the short reads from NGS data (http://soap.genomics.org.cn). In order to successfully de novo assemble the short reads, we filtered the low‐quality reads (When the quality value of a base was less than or equail 7, we defined the base as low‐quality base. If there was more than 10% low‐quality base in a read, the read was referred as low‐quality read.), and used the high‐quality clear data, and used –K, –R, –F, and –u as the program parameters in the SOAP to finish assembly. Each read that was identified as mtDNA was aligned to mtDB. These alignments were then merged, and each alignment column was examined to determine the majority base, yielding large‐assembled contigs or scaffolds of mtDNA. To evaluate accuracy and completeness, the assembled mitochondrial genome sequence homology analysis was conducted using online BlastN in GenBank. Mitochondrial genomes were annotated in reference to the *R. roxellana* mtDNA sequence (JQ821835.1). If the reading frame of protein‐coding genes was disrupted, the original read assembly was revised and corrected manually.

### 2.4 Genetic divergence

To estimate the genetic divergence of the SNJ *R. roxellana* in base composition, we performed the genomic analyses including base composition, base substitution, amino acid replacement with respect to reference mtDNA sequence, and the number of diversity base and the gaps to *R. brelichi* mtDNA sequence using the complete mitochondrial sequence data. Hence, alignment between SNJ *R. roxellana* mtDNA and QL *R. roxellana* mtDNA (reference sequence) was conducted. Alignment between QL *R. roxellana* mtDNA and *R. brelichi* mtDNA, alignment between SG *R. roxellana* mtDNA and *R. brelichi* mtDNA, alignment between SNJ *R. roxellana* mtDNA and *R. brelichi* mtDNA were performed. All pairs’ alignment was conducted using online BlastN in GenBank, and amino acid replacement was analyzed according to gene annotation in GenBank. We further analyzed the genetic divergence between SNJ *R. roxellana* and reference mtDNA based on 13 functional mtDNA genes.

### 2.5 Phylogenetic reconstruction of genus *Rhinopithecus*

To assess the phylogenetic position of SNJ *R. roxellana* among the *Rhinopithecus* genus and the relationships within the *R. roxellana* species, multiple alignments and phylogenetic analyses were conducted using the neighbor‐joining (NJ), maximum‐likelihood (ML), and Bayesian inference (BI) methods. NJ and ML phylogenetic trees were constructed using the MEGA6.0 program (Kimura, 1980; Tamura, Stecher, Peterson, Filipski, & Kumar, 2013). The Kimura 2‐parameter nucleotide model was employed to delete pairwise gaps in the NJ analysis. The reliability of tree topologies was evaluated with 10,000 bootstrap replicates in the NJ analysis and 1,000 bootstrap replicates in the ML analysis. The appropriate DNA substitution model was identified by the lowest Bayesian information criterion scores and calculated using Modeltest version 3.06. BI phylogenetic trees were constructed using BEAST Software (Drummond, Suchard, Xie, & Rambaut, 2012). The Markov chain Monte Carlo algorithm was run for 2 × 1,000,000 generations with four incrementally heated chains. The analyses started with random trees and were sampled every 1 × 1,000 generations. The first 5 × 10,000 trees were treated as burnt in and discarded. The Bayesian consensus tree was constructed using the remaining trees. Internodes with posterior probabilities of 95% were considered statistically significant. In all analyses, positions containing gaps and missing data were eliminated in the phylogenetic tree construction. In this study, eleven complete mtDNA sequences of seven *Rhinopithecus*, one *Pygathrix nigripes*, one *Nasalis larvatus*,* Trachypithecus germaini*, and *Presbytis melalophos* were retrieved from GenBank Database (Table 2); the latter four species were used as outgroups.

**Table 2:** The information on sequences constructing phylogenetic tree

Acc. no.	Species and sequence information	Abbreviation
KM504390.1	SNJ *Rhinopithecu roxellana* mitochondrion, complete genome	SNJ *R. roxellana*
DQ355300.1	SG *Pygathrix roxellana* mitochondrion, complete genome	SG *R. roxellana*
JQ821835.1	QL *R. roxellana* mitochondrion, complete genome	QL *R. roxellana*
JQ821836.1	*Rhinopithecus brelichi* mitochondrion, complete genome	*R. brelichi*
HM125579.1	*Rhinopithecus bieti* mitochondrion, complete genome	*R. bieti*
JQ821838.1	*Rhinopithecus strykeri* mitochondrion, complete genome	*R. strykeri*
HM125578.1	*Rhinopithecus avunculus* mitochondrion, complete genome	*R. avunculus*
JF293094.1	*Nasalis larvatus* mitochondrion, complete genome	*N. larvatus*
JQ821840.1	*Pygathrix nigripes* mitochondrion, complete genome	*P. nigripes*
HQ149047.1	*Trachypithecus germaini* mitochondrion, complete genome	*T. germaini*
DQ355299.1	*Presbytis melalophos* mitochondrion, complete genome	*P. melalophos*

### 2.6 Divergence age analysis

To estimate *R. roxellana* divergence ages, we inferred the evolutionary history based on complete mtDNA, employing a complete molecular clock approach as implemented in MEGA6 (Tamura et al., 2013). In order to ensure a relatively accurate genetic divergence time, we first analyzed the information site and genetic distance of all sequences and then used the NJ and ML methods to infer the evolutionary history (Tamura, Nei, & Kumar, 2004; Tamura et al., 2012). All positions containing gaps and missing data were eliminated, and evolutionary analyses were conducted in MEGA6. In the NJ method, the evolutionary distances are computed using the ML method (Felsenstein, 1985) and are measured as the number of base substitutions per site. The percentage of replicate trees in which associated taxa clustered together in the bootstrap test (1,000 replicates) is shown next to the branches (Tamura et al., 2012). The tree is drawn to scale, with branch lengths in the same units as those of the evolutionary distances that are used to infer the phylogenetic tree. Divergence times for all branching points in the topology were calculated with the RelTime method (Tamure et al., 2012) using the branch lengths contained in the inferred tree. In the ML method, initial tree(s) for the heuristic search were obtained automatically by applying the NJ and BioNJ algorithms to a matrix of pairwise distances estimated to use the maximum composite likelihood approach and then selecting the topology with the superior log likelihood value. The timetree that is shown was also generated using the RelTime method (Tamure et al., 2012). Divergence times for all branching points in the topology were calculated using the ML method based on the Tamura–Nei model (Tamure et al., 2012). Bars around each node represent 95% confidence intervals, which were computed using the method described by Tamure et al (2012). The estimated log likelihood value of the topology is shown. The tree is drawn to scale, with branch lengths measured in the relative number of substitutions per site. We selected the proposed split for the divergence between SG *R. roxellana* (DQ355300.1) and *P. nigripes* of 6.57 (6.69–6.45) million year ago (MYA, Ma) as the calibration points in two models (Perelman et al., 2011).

## 3 RESULTS AND ANALYSIS

### 3.1 Assembly and annotation on mtDNA of SNJ *R. roxellana*

We resequenced the mtDNA of SNJ *R. roxellana* and obtained 217.09 Mb of raw data and 226.95 Mb of raw data from Illumina‐Pipeline. The sequencing depth was 13,568× and 14,184.4×, which was calculated on a base of 16 Kb. After filtering, we got 192.14 Mb of clean data and 200.28 Mb of clean data with coverage of 12,008.8× and 12,517.5×, respectively. Finally, two scaffolds were de novo assembled; one was named N50 (Scaffold 1) with a length of 15,768 bp, and the other was named N90 (Scaffold 2) with the length of 848 bp. The total length was 16,616 bp, but these two scaffolds could not align into a complete genome. In order to link the gap between Scaffolds 1 and Scaffold 2, we designed primers MT_(15,928–16,543)_ and Mt_(7–535)_ according to *R. roxellana* reference mtDNA (GenBank acc. no.: JQ821835.1) to amplify the remaining sequence segment. We got 615‐bp and 528‐bp fragments by PCR amplification and Sanger sequencing. Finally, we got the complete mtDNA genome of the SNJ *R. roxellana* with 16,552 bp by merging sequences with overlapping alignment. The sequence was deposited in GenBank (acc. no.: KM504390.1). The results of the homologous alignment showed that the value of query coverage and identification between the mtDNA sequences of SNJ *R. roxellana* and either *R. roxellana* geographic population (GenBank acc. no: DQ355300.1, SG and JQ821835.1, QL) was 99% and 100%, respectively. These results confirmed the correctness and completeness of the SNJ *R. roxellana* mtDNA sequence assembly. Finally, the same genes as the *R. roxellana* mtDNA sequence (JQ821835.1) were annotated in the complete mtDNA genome of SNJ *R. roxellana*, including 13 protein‐coding genes (ATP6, ATP8, COI‐III, ND1‐6 and 4L, and Cytb), 22 transfer RNA genes (tRNAs), 2 ribosomal RNA genes (12S and 16SrRNA), and two noncoding regions.

### 3.2 Genetic divergence

Alignment of the consensus SNJ *R. roxellana* mtDNA sequence with the *R. roxellana* mtDNA reference sequence (GenBank acc. no.: JQ821835.1) exhibited a total of 73 variable nucleotide positions, consisting of 62 transitions (C‐to‐T = 36, 12 A‐to‐G = 26), six transversions (C‐to‐A = 3, C‐to‐G = 2, T‐to‐A = 1), and five in dels (Table 3). These results confirmed the strong transitional bias (transitions > transversions) in primate mtDNA sequences as reported in the previous study (Zhang, 1998). A total of 19 of these 68 nucleotide substitutions (27.94%) occurred within the noncoding control region of the mtDNA genome. Of the remaining 49 substitutions (72.06%), 37 (54.41%, mean = 4.53%) occurred in 12 of the 13 mitochondrial protein‐coding genes, five (7.35%, mean = 0.33%) occurred in the 22 mitochondrial *tRNA* genes, and seven (10.29%, mean = 5.15%) occurred in the two mitochondrial *rRNA* genes (*12S* and *16S*). All the results indicate that the noncoding control region appears to be the region with the fastest evolution speed, with the protein‐encoding genes ranked second and the *tRNAs* exhibiting slow evolution. Next, we compared the substitutions of 13 protein‐coding genes in SNJ *R. roxellana* with those in the reference *R. roxellana*. Only one gene (*ATP8*) did not show substitutions, and four genes did not have nonsynonymous substitutions. Substitutions of the two protein‐encoding genes (*ND1* and *ND5*) resulted in two amino acids replacement, and substitutions of six protein‐encoding genes led to one amino acid replacement (Table 3). The effect of natural selection during protein evolution can be expressed using the ratio of non‐synonymous mutation (dN) and synonymous mutation (dS). Low dN/dS (<1), high dN/dS (>1), and dN/dS (=1) signify purifying selection, positive selection, and neutral selection, respectively. Overall, *ND1* showed positive selection, *ND2*,* COX*I, and *COX*II displayed neutral selection, and the others exhibited purifying selection. The base composition of SNJ *R. roxellana* mtDNA was different from that of SG *R. roxellana* and QL *R. roxellana* mtDNA, which occurred between A and G base pairs (Table 4). In addition, the genetic divergence between *R. roxellana* and *R. brelichi* demonstrated that SNJ *R. roxellana* had the highest number (766) of divergence bases and fewer gaps (11; Table 5).

**Table 3:** Number of synonymous and nonsynonymous substitutions and amino acid replacement

Gene	Synonymous	Nonsynonymous	Amino acid replacement	dN/dS
*ND1*	1	2	T‐M/A‐T	2
*ND2*	1	1	T‐A	1
*COXI*	1	1	I‐V	1
*COXII*	1	1	A‐G	1
*ATP8*	0	0	—	—
*ATP6*	2	0	—	—
*COXIII*	2	1	Y‐H	0.5
*ND3*	2	0	—	—
*ND4L*	1	0	—	—
*ND4*	5	1	T‐A	0.2
*ND5*	6	2	L‐F/V‐A	0.333333
*ND6*	1	0	—	—
*CTYb*	4	1	N‐S	0.25

*COXI–III*, cytochrome *c* oxidase subunit 1–3; *ATP6* and *ATP8*, two subunits of ATP synthase; *ND1–6* and *4L*, NADH dehydrogenase subunits 1–6 and 4L; *Cytb*, cytochrome b.

**Table 4:** The difference in base pairs of mtDNA of *Rhinopithecu roxellana* mtDNA

	T(U)	C	A	G	Total
*SNJ R. roxellana*	30.4	25.9	32.1	11.6	16,552
*SG R. roxellana*	30.4	25.9	32	11.7	16,549
*Ql R. roxellana*	30.4	25.9	32	11.7	16,551

**Table 5:** Analyses of genetic divergence of *Rhinopithecu roxellana* mtDNA

Alignment sequences	Divergence base number (%)	Gap number
DQ355300.1–JQ821836.1	753 (4.55)	12
JQ821835.1–JQ821836.1	756 (4.57)	12
KM504390.1–JQ821836.1	766 (4.63)	11

### 3.3 Phylogenetic analyses

**Figure 2:**
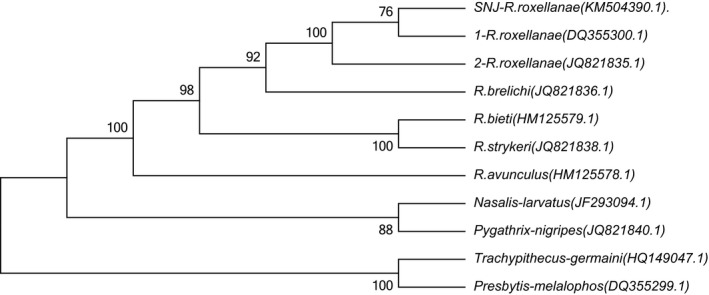
Evolutionary relationships of *Rhinopithecus* genus was inferred using the neighbor‐joining method. The evolutionary distances were computed using the maximum composite likelihood method and are shown as the number of base substitutions per site

In this study, after all the positions containing gaps and missing data were eliminated, 16,460 positions were used to construct the phylogenetic tree. Regardless of the phylogenetic method employed, the topology structure of three phylogenetic trees was highly congruent, so we only present the NJ tree here (Figure 2). All analyses indicated that the *Rhinopithecus* genus possessed a monophyletic origin and *R. avunculus* diverged first, *R. bieti* and *R. strykeri* formed sister taxa and diverged second, and *R. roxellana and R. brelichi* formed sister taxa and diverged last. The phylogenetic tree shows *R. roxellana* has a monophyletic origin and SNJ *R. roxellana* diverged at the latest among *R. roxellana* geographical populations.

### 3.4 Divergence age analysis

**Figure 3:**
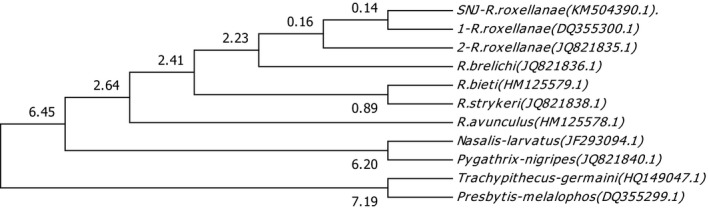
Evolutionary relationships of timetree. The evolutionary history was inferred using the neighbor‐joining method. Divergence times were showed in the branch in the topology tree

**Figure 4:**
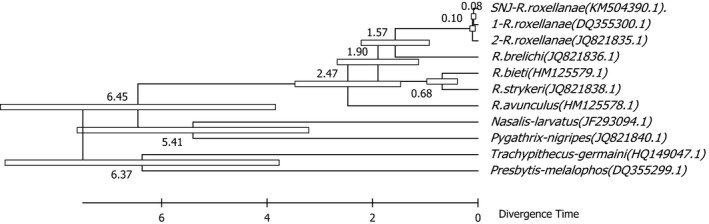
Molecular phylogenetic analysis was conducted by maximum‐likelihood method (timetree). Divergence times were showed on the branch in the topology tree

Divergence times for all branching points in the topology were calculated with the RelTime method, and all branching points in the topology were calculated using the NJ method and the ML method based on the Tamura–Nei model. Codon positions included 1st + 2nd + 3rd, using the branch lengths contained in the inferred tree. In the NJ methods, the optimal tree with the sum of branch length was 0.47216046. In the ML methods, the estimated log likelihood value of the topology was −57,223.6675. In the NJ method, the divergence time of *Rhinopithecus* genus was from ca. 2.23 to 2.64 Ma, *R. roxellana and R. brelichi* diverged in ca. 2.23 Ma, *R. bieti* and *R. strykeri* diverged in ca. 0.89 Ma, *R. avunculus* separated from genus *Rhinopithecus* in ca. 2.64 Ma, and divergence time of SNJ *R. roxellana* was ca. 0.14 Ma. The most recent common ancestor of *R. roxellana* lived ~0.16 Ma (Figure 3). In the ML method, the divergence time of genus *Rhinopithecus* was from ca. 1.57–2.47 Ma, *R. roxellana* and *R. brelichi* diverged in ca. 1.57 Ma, *R. bieti and R. strykeri* diverged in ca. 0.68 Ma, *R. avunculus* separated from genus *Rhinopithecus* in ca. 2.47 Ma, and divergence time of SNJ *R. roxellana* was ca. 0.08 Ma. The most recent common ancestor of *R. roxellana* lived approximately 0.10 Ma (Figure 4).

## 4 DISCUSSION

### 4.1 Evolutionary relationship of geographic groups of *R. roxellana*

The phylogenetic relationships of the *Rhinopithecus* genus have been studied (Chang, Liu, Yang, et al., 2012; Karanth et al., 2008; Li et al., 2010; Osterholz et al., 2008; Yang et al., 2012); however, the evolutionary relationships of *R. roxellana* are still in dispute (Pan et al., 2009). Some researchers insisted that the QL and SNJ populations originated from the SG population (Chang, Liu, Yang, et al., 2012; Li et al., 2007; Luo et al., 2012), while others believed that the SG population was a heterozygosis of the QL and SNJ populations (Chang, Luo, Liu, et al., 2012; Liedigk et al., 2012). The Shennongjia National Nature Reserve is the most eastern habitat of *R. roxellana* in China, and SNJ *R. roxellana* has the smallest population (Pan et al., 2009). Analyses of the complete mtDNA control region suggested that the QL and SNJ populations originated from the SG population, but there was no gene flow between the SNJ and SG populations (Luo et al., 2012). In contrast, the structure analyses of 16 microsatellite loci showed that the SNJ population consisted of two groups and that the SNJ population originated from the SG and QL populations (Chang, Liu, Yang, et al., 2012). Moreover, some studies showed SNJ population had the lowest diversity (Chang, Luo, Liu, et al., 2012; Chang, Liu, Yang, et al., 2012; Luo et al., 2012), another study showed SNJ population had rich genetic diversity (He et al., 2010). Therefore, the origin of *R. roxellana* is controversial in previous studies because of different genetic markers. In this study, we used three complete mitochondrial genome sequences, instead of the complete mtDNA control region (D‐loop), to assess the origin of *R. roxellana* by analyzing the genetic divergence,divergence ages and constructing the phylogenetic tree. The result showed SNJ *R. roxellana* was well clustered together with other two *R. roxellana* in the phylogenetic tree, and SNJ *R. roxellana* diverged at the latest among *R. roxellana* geographical populations. Although SNJ *R. roxellana* had the richest genetic divergence in gene composition, and SNJ *R. roxellana* was well clustered together with other two *R. roxellana* in the phylogenetic tree, the bootstrap value (76) of phylogenetic tree between SNJ *R. roxellana* and other two *R. roxellana* was low. These results indicated SNJ *R. roxellana* was a newest rapid evolutionary geographic population of *R. roxellana*, which was similar with recently report (Zhou et al., 2016). The result, to some extent, could explain why the early results were controversial.

### 4.2 The divergence ages of geographic groups of *R. roxellana*

The divergence ages of endangered species motivate us to determine their protected status and inform protection policy. The early study on the base of autosomal genome clusters showed the divergence ages between the northern species (*R. roxellana* and *R. brelichi*) and the “Himalayan” species (*R. bieti* and *R. strykeri*) was 1.60 Ma, and the divergence ages between *R. roxellana* and *R. brelichi* was 1.69 Ma (Zhou et al., 2014). But the result on the base of mitochondrial haplotype clusters showed the divergence ages of geographic of *R. roxellana* were 1.17 ± 0.70 and 0.53 ± 0.30 Ma (Chang, Luo, Liu, et al., 2012). In the study, although the evolutionary tendency of the *Rhinopithecus* genus and *R. roxellana* was consistent in the NJ and ML methods, the variation in divergence times was greater in ML than in NJ, and the divergence times occurred later in ML than in NJ. In the NJ method, the divergence ages between the northern species and the “Himalayan” species were 2.41 Ma, and the divergence ages between *R. roxellana* and *R. brelichi* were 2.23 Ma, and the divergence time of the *R. roxellana* was from ca. 0.14–1.16 Ma, and that of SNJ *R. roxellana* was ca. 0.14 Ma. In the ML method, the divergence ages between the northern species and the “Himalayan” species were 1.9 Ma, and the divergence ages between *R. roxellana* and *R. brelichi* were 1.57 Ma, and the divergence time of the *R. roxellana* was from ca. 0.08–0.1 Ma, and that of SNJ *R. roxellana* was ca. 0.08 Ma. Because the divergence ages between *R. roxellana* and *R. brelichi* were 1.57 Ma in ML methods, which was coincide with the uplift of the Tibetan plateau—Yuanmu movement in approximately 1.6 million years ago; and the divergence time of the *R. roxellana* was from ca. 0.08–0.1 Ma in ML methods, which agreed with the penultimate glaciation (0.13–0.3 million years ago; Zhou et al., 2016). So we thought the divergence ages of *R. roxellana* acquired from ML methods were closer to reality.

### 4.3 Functional genes and environmental adaptability of mtDNA

Mitochondria play important roles in generating energy by oxidative phosphorylation and taking part in energy metabolism. The oxidative phosphorylation system consists of 13 essential proteins encoded by corresponding genes, including *ATP*_*6*_, *ATP*_*8*_, *COI‐III*,* ND*_*1‐6*_ and *4L*, and *Cytb*. These 13 genes are particularly used as effective genetic markers to investigate the molecular basis of organismal adaptation to environments (Fonseca, Johnson, O'Brien, Ramos, & Antunes, 2008; Zheng, Xu, & Shen, 2002). Previous studies showed that cytochrome c oxidase genes of Ca*prini antelope*, camelids and Tibetan antelope (Di, Zambelli, & Rioja, 2009; Hassanin, Ropiquet, Couloux, & Cruaud, 2009; Ning, Xiao, Li, Hua, & Zhang, 2010), NADH dehydrogenase genes of Tibetan horses (Xu et al., 2005), cytochrome b geneand ATP synthase of alpacas (Xu et al., 2007) went through adaptive evolution. The *ND*_*2*_ and *ND*_*6*_ of *R. roxellana* presented significantly positive adaptation to high altitude and cold weather stress. In the study, 1 gene (*ND*_*1*_) showed positive selection, three genes (*ND*_*2*_, *COXI*, and *COXII*) displayed neutral selection, and the others exhibited purifying selection in the complete mitochondrial genome sequence of SNJ *R. roxellana*. These results obviously indicated more purifying selections on mitochondrial proteins in SNJ *R. roxellana*, consistent with strong purifying selections on mitochondrial proteins in primates (Felsenstein, 1985). Purifying selections was also known as negative selections, that is, more unfavorable environmental factors acted on SNJ *R roxellana*. The more unfavorable environmental factors resulted in more abundant genetic divergence and less similarity.

## 5 CONCLUSIONS

We obtained the complete mitochondrial genome sequence of SNJ *R. roxellana* with a total length of 16,552 bp by NGS and Sanger sequencing. The analyses on genetic composition, phylogenetic tree, and divergence time showed that SNJ *R. roxellana* mtDNA had the most genetic divergence, the least structure, and the more recent divergence times of *R. roxellana*, which indicated SNJ *R. roxellana* was a newest rapid evolutionary geographic population of *R. roxellana* and should be protected as a unit. Our findings are more comprehensive and promote the development of a more accurate protection policy for SNJ *R. roxellana*.

## CONFLICT OF INTEREST

None declared.
